# MDM2 inhibitor APG-115 exerts potent antitumor activity and synergizes with standard-of-care agents in preclinical acute myeloid leukemia models

**DOI:** 10.1038/s41420-021-00465-5

**Published:** 2021-05-03

**Authors:** Douglas D. Fang, Qiuqiong Tang, Yanhui Kong, Tao Rong, Qixin Wang, Na Li, Xu Fang, Jiaxing Gu, Dengkun Xiong, Yan Yin, Jing Deng, Dajun Yang, Yifan Zhai

**Affiliations:** 1 Ascentage Pharma (Suzhou) Co., Ltd., 218 Xinghu Street, Suzhou, Jiangsu Province China; 2 Department of Experimental Research, State Key Laboratory of Oncology in South China, Collaborative Innovation Center for Cancer Medicine, Sun Yat-Sen University Cancer Center, Guangzhou, China

**Keywords:** Leukaemia, Cancer

## Abstract

Acute myeloid leukemia (AML) is a clinically and genetically heterogeneous clonal disease associated with unmet medical needs. Paralleling the pathology of other cancers, AML tumorigenesis and propagation can be ascribed to dysregulated cellular processes, including apoptosis. This function and others are regulated by tumor suppressor P53, which plays a pivotal role in leukemogenesis. Opposing P53-mediated activities is the mouse double minute 2 homolog (MDM2), which promotes P53 degradation. Because the *TP53* mutation rate is low, and MDM2 frequently overexpressed, in patients with leukemia, targeting the MDM2-P53 axis to restore P53 function has emerged as an attractive AML treatment strategy. APG-115 is a potent MDM2 inhibitor under clinical development for patients with solid tumors. In cellular cultures and animal models of AML, we demonstrate that APG-115 exerted substantial antileukemic activity, as either a single agent or when combined with standard-of-care (SOC) hypomethylating agents azacitidine (AZA) and decitabine (DAC), or the DNA-damaging agent cytarabine (Ara-C). By activating the P53/P21 pathway, APG-115 exhibited potent antiproliferative and apoptogenic activities, and induced cell cycle arrest, in *TP53* wild-type AML lines. In vivo, APG-115 significantly reduced tumor burden and prolonged survival. Combinations of APG-115 with SOC treatments elicited synergistic antileukemic activity. To explain these effects, we propose that APG-115 and SOC agents augment AML cell killing by complementarily activating the P53/P21 pathway and upregulating DNA damage. These findings and the emerging mechanism of action afford a sound scientific rationale to evaluate APG-115 (with or without SOC therapies) in patients with AML.

## Introduction

Acute myeloid leukemia (AML) is a genetically and clinically heterogeneous clonal disease characterized by the rapid proliferation of immature myeloid progenitors, with suppression of hematopoiesis^1,2^. Allogeneic hematopoietic stem cell transplantation (allo-HCT) remains the most promising modality to effect cure. However, allo-HCT can be offered only to a minority of younger, fit patients, whereas AML primarily affects individuals older than 60 who are hence not suitable candidates^3^. Patients who have AML and are ineligible for allo-HCT and/or harbor high-risk karyotypes typically have poor outcomes, with an estimated median overall survival of only 1 year^4^.

For elderly patients with AML, the DNA hypomethylating agents azacytidine (AZA; 5-azacytidine) and decitabine (DAC; 5-aza-2′-deoxycytidine) have become standards of care^5–8^; however, rates of CR, together with CR and incomplete cell-count recovery in patients with newly diagnosed or advanced AML remains low, typically range from only 20 to 30%^9^.

The regimen of choice for induction in many patients with newly diagnosed AML is the DNA-damaging agent cytarabine (Ara-C), administered daily for 7 days, followed by daunorubicin for 3 days^10,11^. This so-called 7 + 3 regimen has been associated with high drug resistance and a low 5-year OS rate^12,13^. Even with recently approved molecularly targeted agents, the 5-year survival rate in patients younger than 65 with AML is only about 45%^14^. In addition, the number of newly diagnosed AML cases continues to rise, at an annual rate of 2% from 2007 to 2016^15^. There is hence an urgent need to identify novel agents that enhance clinical outcomes in AML.

Evasion of apoptosis is a pathological hallmark of AML and other cancers^3^. Apoptosis and multiple other cellular functions are mediated by the tumor suppressor P53, which plays a pivotal role in leukemogenesis^16^. P53 is precisely controlled by the negative regulator mouse double minute 2 homolog (MDM2), which blocks transcriptional activity of P53 and promotes its proteasomal degradation. In various cancers, MDM2 overexpression is a prominent mechanism to impair P53 function. Studies of AML suggest that the *TP53* mutation rate remains as low as 10%^17–20^, while MDM2 is frequently overexpressed^21–23^, rendering the P53-MDM2 axis an attractive pharmacologic target for small-molecule inhibitors.

In addition to monotherapy, combinations of MDM2 inhibitors with SOC agents could improve antileukemic efficacy^24^. A phase I/Ib clinical trial demonstrated that MDM2 inhibitor idasanutlin (RG-7388) combined with Ara-C conferred durable clinical efficacy, including CR in patients with r/r AML^25^. However, mechanisms underlying synergy between these therapies and SOC agents (e.g., DAC, AZA, and Ara-C) have yet to be elucidated.

We have developed a novel, potent MDM2 inhibitor (APG-115), which is being evaluated in patients with solid tumors (NCT02935907, NCT03611868). However, the effects of this small-molecule agent when administered with SOC and other treatments for AML are unknown. To address this knowledge gap, we established the following objectives for this study: (1) to evaluate the antileukemic activity of APG-115, administered alone or combined with AZA, DAC, or Ara-C, in preclinical models of AML; and (2) to assess synergy of APG-115 combined with these SOC therapies, and, if present, formulate underlying molecular mechanisms.

## Results

### APG-115 potently inhibits cellular proliferation and induces apoptosis and cell cycle arrest in *TP53* wild-type AML cell lines

**Fig. 1:**
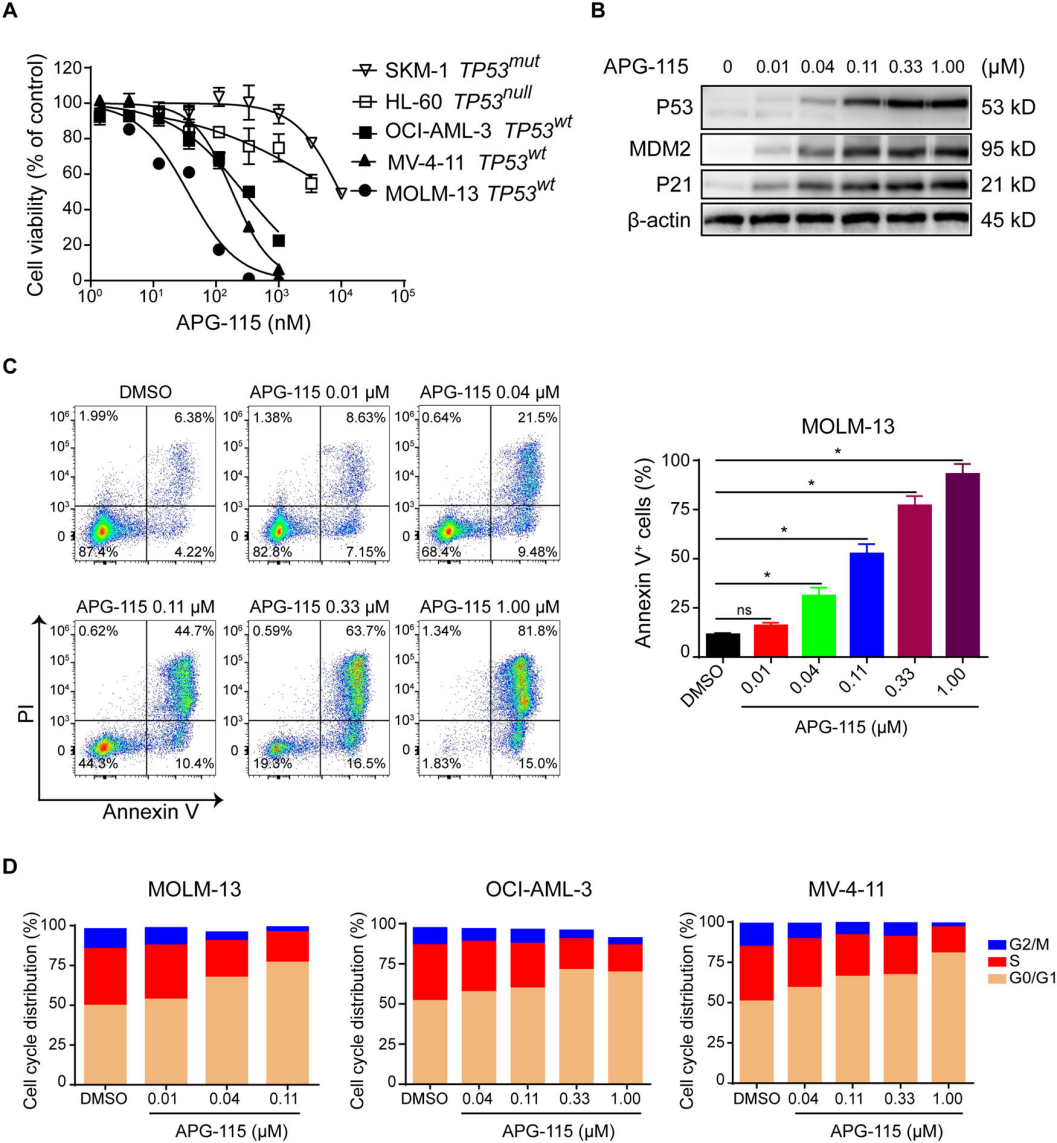
APG-115 potently inhibits cellular proliferation and induces apoptosis, with G_0_/G_1_ cell cycle arrest, in *TP53*^*wt*^ AML cell lines. **A** Mean (SD) cellular viability of MOLM-13 (*TP53*^*wt*^), MV-4-11 (*TP53*^*wt*^), OCI-AML-3 (*TP53*^*wt*^), HL-60 (*TP53*^*null*^), and SKM-1 (*TP53*^*mut*^) cells treated with increasing concentrations of APG-115 for 72 h (*n* = 3 replicates). **B** Total protein levels of MDM2, P53, P21, and the loading control β-actin by Western blot in MOLM-13 cells exposed to APG-115 at increasing concentrations for 4 h. **C** Representative flow cytometry results from two independent experiments (*left panel*) and mean (SD) percentage of MOLM-13 cells in apoptosis (*right panel*) presented as *n* = 4 technical replicates. *p < 0.0001; ns not significant; compared to dimethyl sulfoxide vehicle (DMSO) via two-tailed Student *t* tests for data from three independently repeated experiments. **D** Increased cell cycle arrest in the G_0_/G_1_ phase, as indicated by flow cytometry in AML cells exposed to increasing concentrations of APG-115 for 48 h. Data shown are representative images of two independent experiments.

The in vitro antiproliferative activity of APG-115 was first evaluated in a panel of five distinct AML cultures, including three *TP53* wild-types *(TP53*^*wt*^; MOLM-13, MV-4-11, OCI-AML-3), a *TP53* null (*TP53*^*null*^*;* HL-60), and a *TP53* mutant (*TP53*^*mut*^; SKM-1) cell lines (Fig. 1A). HL-60 is a cell line with documented *TP53* gene deletion in both alleles, while SKM-1 is a cell line carrying a missense mutation (p.R248Q) in the *TP53* gene. All three *TP53*^*wt*^ cell lines were more sensitive (vs. *TP53*^*mut*^ lines) to APG-115 treatment. Half-maximal inhibitory concentration (IC_50_) values of APG-115 were at least one order of magnitude lower (i.e., more active) in decreasing cellular proliferation in *TP53*^*wt*^, compared to *TP53*^*null*^ and *TP53*^*mut*^ cell lines. Mean (SD) values of IC_50_ for APG-115 were 26.8 (4.9) nM in MOLM-13, 165.9 (42.4) nM in MV-4-11, and 315.6 (97) nM in OCI-AML-3 cells (Supplementary Table 1). Compared to these *TP53*^*wt*^ cells, HL-60 and SKM-1 cells with defected *TP53* were resistant to APG-115 treatment, with mean (SD) IC_50_ values of 2558.3 (581.5) nM in HL-60 cells and 8,947.3 (569.6) nM in SKM-1 cells (Supplementary Table 1). APG-115 was also more potent than MDM2 inhibitor RG-7388 in both *TP53*^*wt*^ (MOLM-13 and MV-4-11) and *TP53*^*mut*^ (SKM-1) AML cell lines (Supplementary Table 1).

In *TP53*^*wt*^ MOLM-13 cells, APG-115 potently and dose-dependently induced rapid accumulation of MDM2 as well as P53 and its downstream protein P21 (Fig. 1B), with minimally effective concentrations of approximately 0.01–0.04 μM. APG-115 also induced dose-dependent cell apoptosis in MOLM-13 cells (Fig. 1C). Treatment with APG-115 for 48 h led to apoptosis in approximately 31% of cells at APG-115 0.04 μM, 55.1% at 0.11 μM, 80.2% at 0.33 μM, and 96.8% at 1 μM (each *p* < 0.0001 vs. DMSO vehicle). Moreover, APG-115 treatment of the three *TP53*^*wt*^ AML cell lines reduced percentages of cells in the S phase, indicating cell cycle arrest in the G_0_/G_1_ phase (Fig. 1D). In the aggregate, these findings demonstrate that APG-115 effectively: (1) activated the P53/P21 pathway, (2) inhibited cellular proliferation, and (3) induced apoptosis and cell cycle arrest.

### APG-115 significantly reduces the leukemic burden and prolongs survival in *TP53* wild-type AML xenograft models

**Fig. 2:**
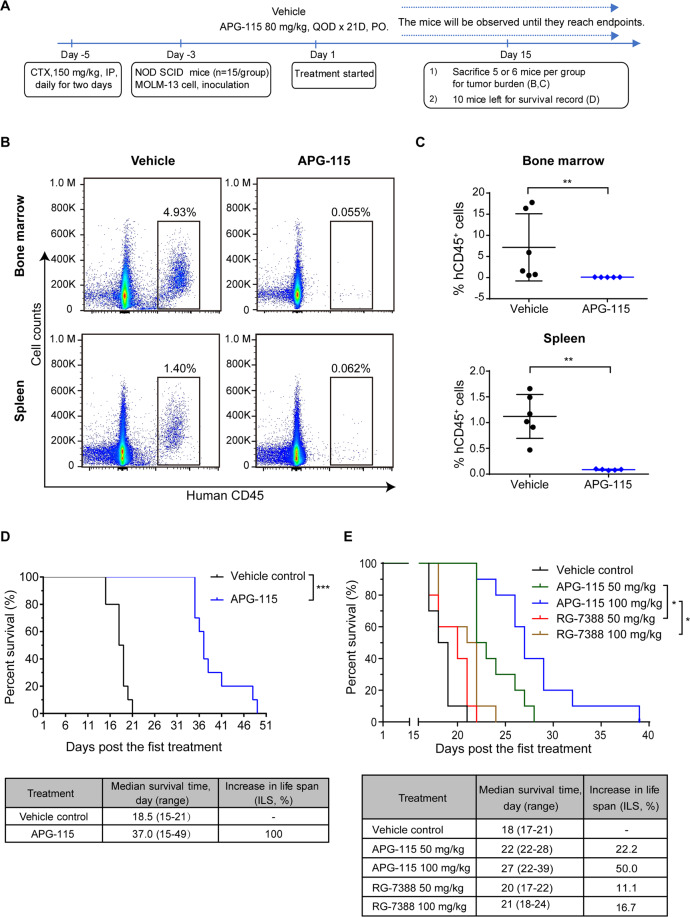
Single-agent APG-115 significantly reduces the leukemic burden and prolongs survival in systemic *TP53*^*wt*^ MOLM-13 AML xenografts. **A** Study schema of the experimental paradigm to study tumor burden and animal survival in the systemic MOLM-13 AML model. For CD45 analysis, mice were treated with APG-115 for 15 days. For survival analysis, mice were treated with APG-115 for 21 days. **B** Representative flow cytometry results show tumor burden (i.e., engraftment of human CD45^+^ cells) in murine femoral bone marrow and spleen. Five or six mice treated as in A from each treatment group were euthanized and examined on Day 15. **C** Significantly lower proportions of human CD45^+^ cells in the bone marrow and spleen collected from mice exposed to APG-115 (treated as in A) compared to vehicle (**p < 0.01 by two-tailed Student tests). **D** Kaplan–Meier proportional-hazards plots show significantly increased percentage survival in mice receiving APG-115 per the dosing conditions shown in A (~50 days) compared to mice receiving vehicle. APG-115 prolonged median survival time by 18.5 days compared to vehicle control (37.0 vs. 18.5 days), *p* < 0.0001 by log-rank test; *n* = 9–10 mice. CTX cyclophosphamide, IP intraperitoneally, NOD SCID nonobese diabetic severe combined immunodeficient, PO orally, QOD every other day. **E** APG-115 confers significantly increased in vivo survival compared to RG-7388 in a systemic AML xenograft model derived from *TP53* wild-type MOLM-13 cells. NOD SCID mice intravenously implanted with 1 × 10^7^ MOLM-13 cells (*n* = 10/group) were treated 3 days after cell inoculation. APG-115 and RG-7388 were orally administered daily for 7 days. Kaplan–Meier curve depicts mouse survival. *p < 0.05 by log-rank test.

Figure 2 depicts the antileukemic activity of APG-115, including effects on survival in a systemic AML xenograft model derived from MOLM-13 cells, including the study schema (Fig. 2A). In brief, APG-115 treatment was initiated 3 days after cellular inoculation and, after 15 days of treatment, five or six mice were randomly selected from each group and euthanized to analyze human CD45^+^ cells and assess tumor burden. Compared to vehicle control, APG-115 treatment substantially depleted the proportion of human CD45^+^ leukemic cells, to <0.1% in both bone marrow (vs. 0.41‒16.7%), and spleen (vs. 0.39‒1.40%; each comparison *p* < 0.005; Fig. 2B, C). The remaining (no euthanized) mice were continually observed for survival rates after suspending the APG-115 dose. The median overall survival of mice in the control group was 18.5 days (range from 15 to 21 days), whereas APG-115 treatment significantly extended median survival time up to 37.0 days (range from 15 to 49 days (*p* < 0.0001, Fig. 2D).

In another independent experiment conducted in the same model, treatment with oral APG-115 consistently exhibited potent, dose-dependent antitumor activities, prolonging median OS to 23 days (range from 22 to 29 days) at an alternate-day dosing schedule of 20 mg/kg for 21 days, 30.5 days (range from 25 to 33 days) at 50 mg/kg once daily for seven days, and 38.5 days (range from 36 to 47 days) at 100 mg/kg once daily for 7 days, compared to 22 days (range from 20 to 22 days) in controls (each *p* < 0.05; Supplementary Fig. S1). Compare to the alternate-day dosing schedule of APG-115 at a clinically relevant dose (i.e., 20 mg/kg) currently used in clinical trials in patients with solid tumors, a pulsed high-dose (i.e., 50 and 100 mg/kg) schedule successfully achieves more substantial effect in the preclinical AML models, indicating that identifying the optimal dose regimen for APG-115 in AML is necessary.

Under the same dosing conditions (PO, QD for 7 days), APG-115 significantly increased median survival compared to RG-7388 in mice bearing MOLM-13 xenografts: by 2 days at equivalent 50 mg/kg doses and by 6 days at 100 mg/kg (each *p* < 0.05 for APG-115 vs. RG-7388, Fig. 2E). These cell-derived-xenograft (CDX) findings extend the foregoing in vitro data, suggesting that APG-115 exerts potent in vivo antileukemic (survival-promoting) activity, which significantly exceeded that of MDM2 inhibitor RG-7388.

### Combinations of APG-115 with AZA, DAC, or Ara-C exhibit synergistic antiproliferative and apoptogenic activity in *TP53*^*wt*^ AML cell lines

**Fig. 3:**
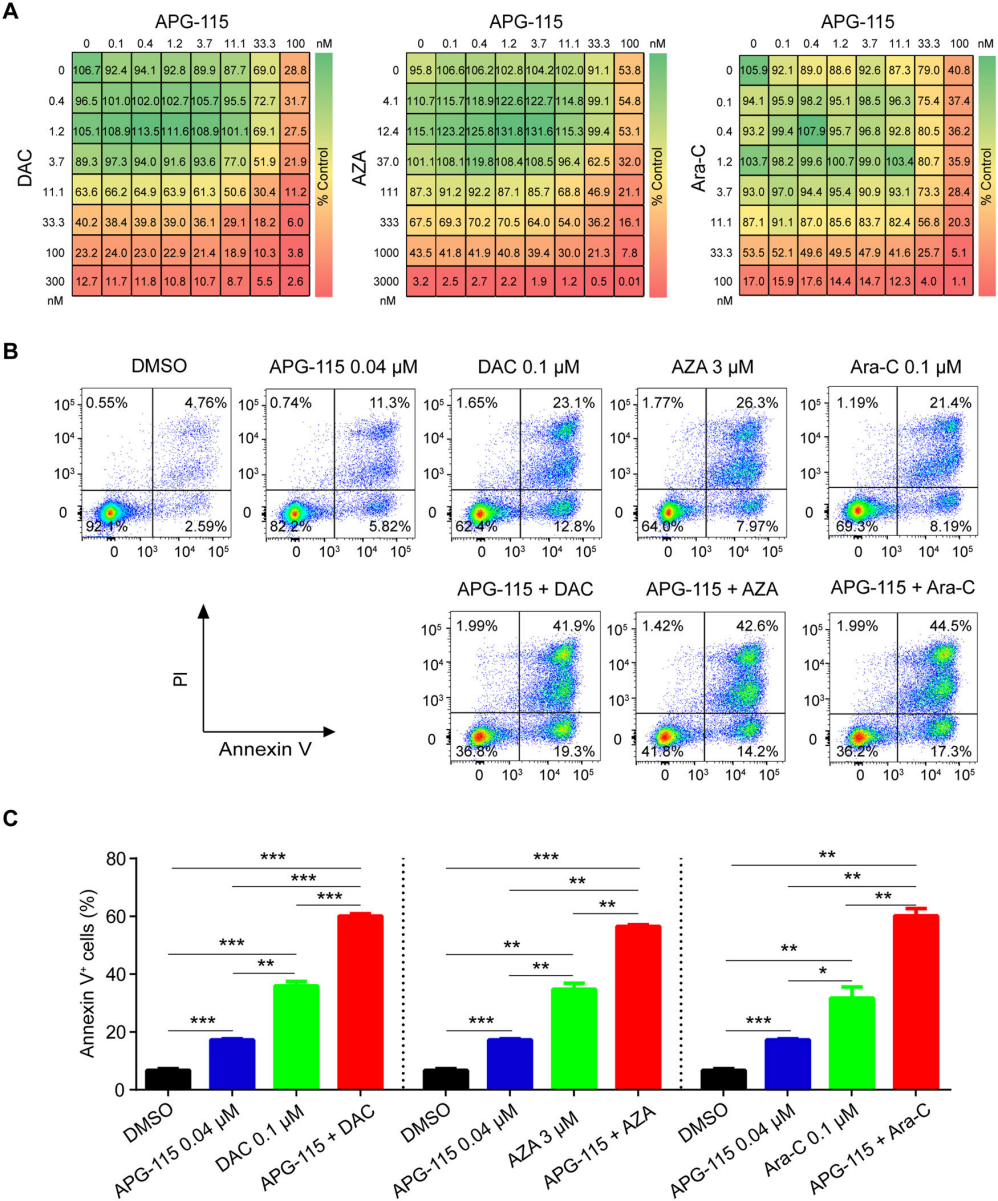
APG-115 synergizes with AZA, DAC, and Ara-C to inhibit cellular growth and induce apoptosis in *TP53*^*wt*^ AML cells. **A** Drug dose matrix of human MOLM-13 cells treated with increasing concentrations of APG-115, AZA, DAC, Ara-C or their combinations for 72 h. Cell viability was assessed using the CellTiter-Glo^®^ assay. Representative results from three independent experiments are presented. The drug dose matrix indicates the percentage of cell viability of treated cells relative to vehicle controls. **B** Representative flow cytometry results from three independent experiments involving MOLM-13 cells treated with indicated concentrations of APG-115, DAC, AZA, and Ara-C (alone or in combination) for 48 h show enhanced apoptogenic effects. **C** Mean (SD) proportions of apoptotic cells (*n* = 3 replicates) show significant increases in cell lines exposed to APG-115 combined with SOC agents (vs. either alone). *p < 0.05, **p < 0.01, ***p < 0.001 by one-way ANOVA tests for data from three independently repeated experiments. DMSO, dimethylsulfoxide (vehicle); PI, propidium iodide.

Antiproliferative activity against *TP53*^*wt*^ MOLM-13 AML cell line was enhanced when APG-115 was combined with AZA, DAC, or Ara-C, as depicted in Fig. 3A. Similar effects were also observed when APG-115 was combined with SOC treatments in MV-4-11 and OCI-AML-3 cell lines (Supplementary Fig. 2).

Combination treatments including APG-115 further enhanced apoptogenic activity. Compared with single agents, the combination of APG-115 with AZA, DAC, or Ara-C led to significant increases in MOLM-13 cell apoptosis (Fig. 3B, C). On the other hand, only single-agent APG-115 consistently induced cell cycle arrest at the G_0_/G_1_ phase; concurrent treatment with APG-115 and concomitant SOC agents did not further enhance this effect on cell cycle arrest (Supplementary Fig. 3).

Collectively, these data suggest that APG-115, combined with the SOC agents AZA, DAC, and Ara-C, synergistically inhibits cellular proliferation and induces apoptosis in *TP53*^*wt*^ AML cells. Given that cell cycle arrest was enhanced by single-agent APG-115, but not incrementally when combined with SOC agents, the antileukemic effects of APG-115 combination treatments are evidently primarily mediated by APG-115 induced inhibition of AML cellular growth and upregulated apoptosis.

### The combination of APG-115 with AZA or DAC exerts superior antileukemic activity in *TP53* wild-type AML xenograft models

**Fig. 4:**
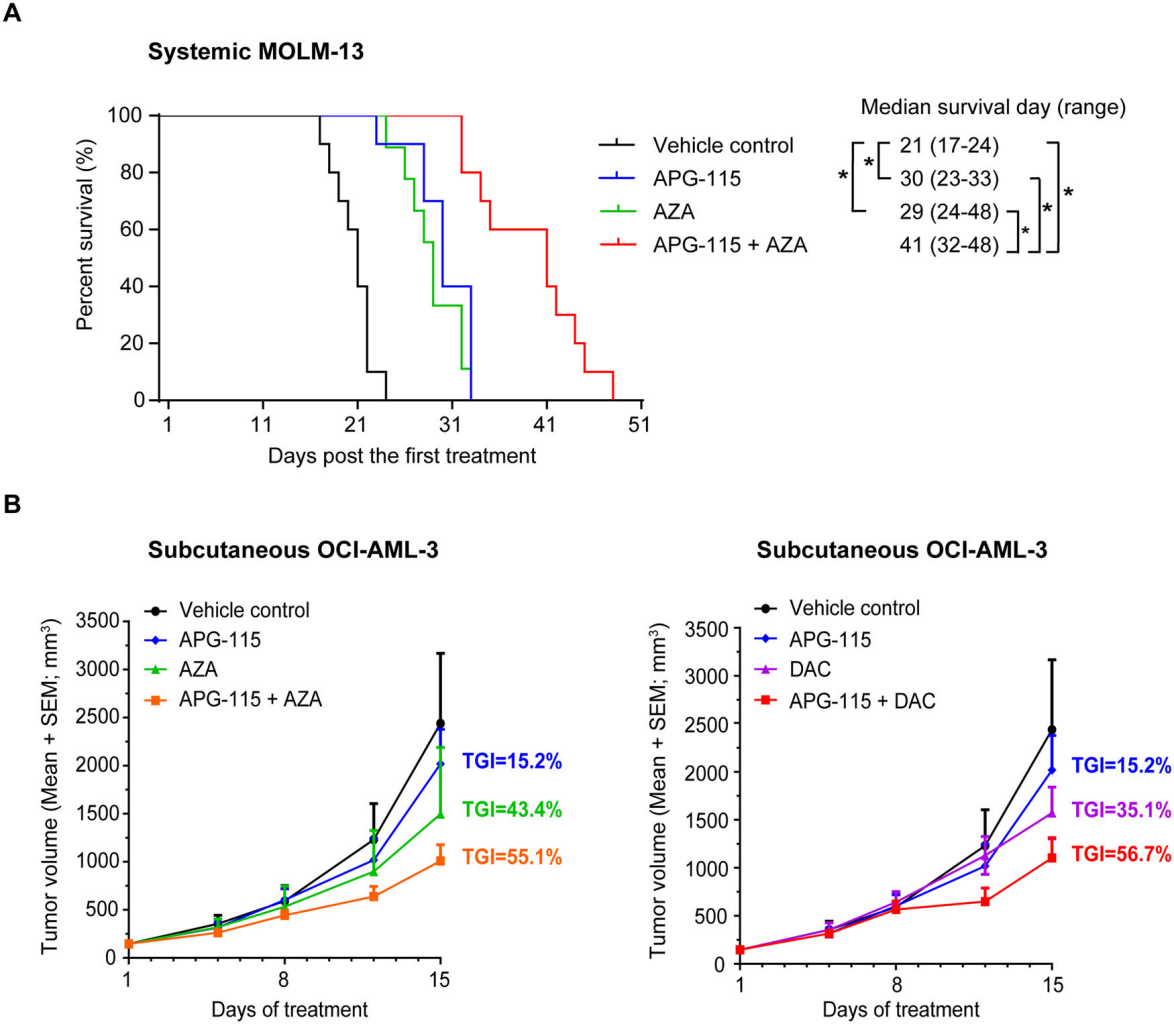
APG-115 enhances the antileukemic activity of AZA or DAC in AML xenograft models. Three days after cell implantation, mice were treated with APG-115 (50 mg/kg, PO, QD), AZA (2 mg/kg, IV, QD), the combination, or vehicle for 7 days. **A** Kaplan–Meier curves show that APG-115 combined with AZA conferred significantly prolonged survival compared to any single agent in a systemic MOLM-13 AML xenograft model of NOD SCID mice (*n* = 10 per group): 41 days with APG-115-AZA compared to 30 days with APG-115, 29 days with AZA, and 21 days with the vehicle (*p < 0.05 for each comparison of APG-115‒Aza with single agents or vehicle by log-rank test). **B** APG-115 potentiates the growth-inhibitory effects of AZA and DAC in NOD SCID mice bearing subcutaneous OCI-AML-3 xenograft tumors. Mice were treated with APG-115 (50 mg/kg, PO, QOD for 15 days), AZA (2 mg/kg, IV, QD for 7 days), DAC (1 mg/kg, IV, QD for 7 days), or combinations as indicated (*n* = 5 per group). Tumor growth inhibition (TGI) was calculated at the endpoint.

When combined with hypomethylating agents, APG-115 exhibited superior in vivo antileukemic activity and augmented life expectancy in AML xenograft models. As shown in Fig. 4A, the median survival time of animals in the vehicle control group was 21 days (range from 17 to 24 days). Either APG-115 or AZA monotherapy significantly prolonged survival in mice bearing systemic MOLM-13 AML xenografts, to median values of 30 days (APG-115, rang from 23 to 33 days) and 29 days (AZA, range from 24 to 48 days, each *p* < 0.05 vs. vehicle control). Compared to either single agents or vehicle control, the combination of APG-115 with AZA significantly extended survival to a median of 41 days (range from 32 to 48 days, *p* < 0.05 vs. single-agent AZA, APG-115, or vehicle).

In a subcutaneous OCI-AML-3 AML xenograft model, APG-115 with SOC agents potentiated tumor growth inhibition (TGI) compared to single agents: 55.1% on APG-115 with AZA, 43.4% with AZA, and 15.2% on APG-115 (each vs. vehicle; Fig. 4B). Comparable TGI values with DAC were 56.7% (APG-115 + DAC), 35.1% (DAC), and 15.2% (APG-115). These findings support the conclusion that APG-115 cooperatively augments antileukemic activity when combined with SOC therapies.

### The combination of APG-115 with either AZA or Ara-C cooperatively activates the P53 pathway and downregulates genes involved in cell cycle progression and mismatch repair in *TP53* wild-type AML cells

**Fig. 5:**
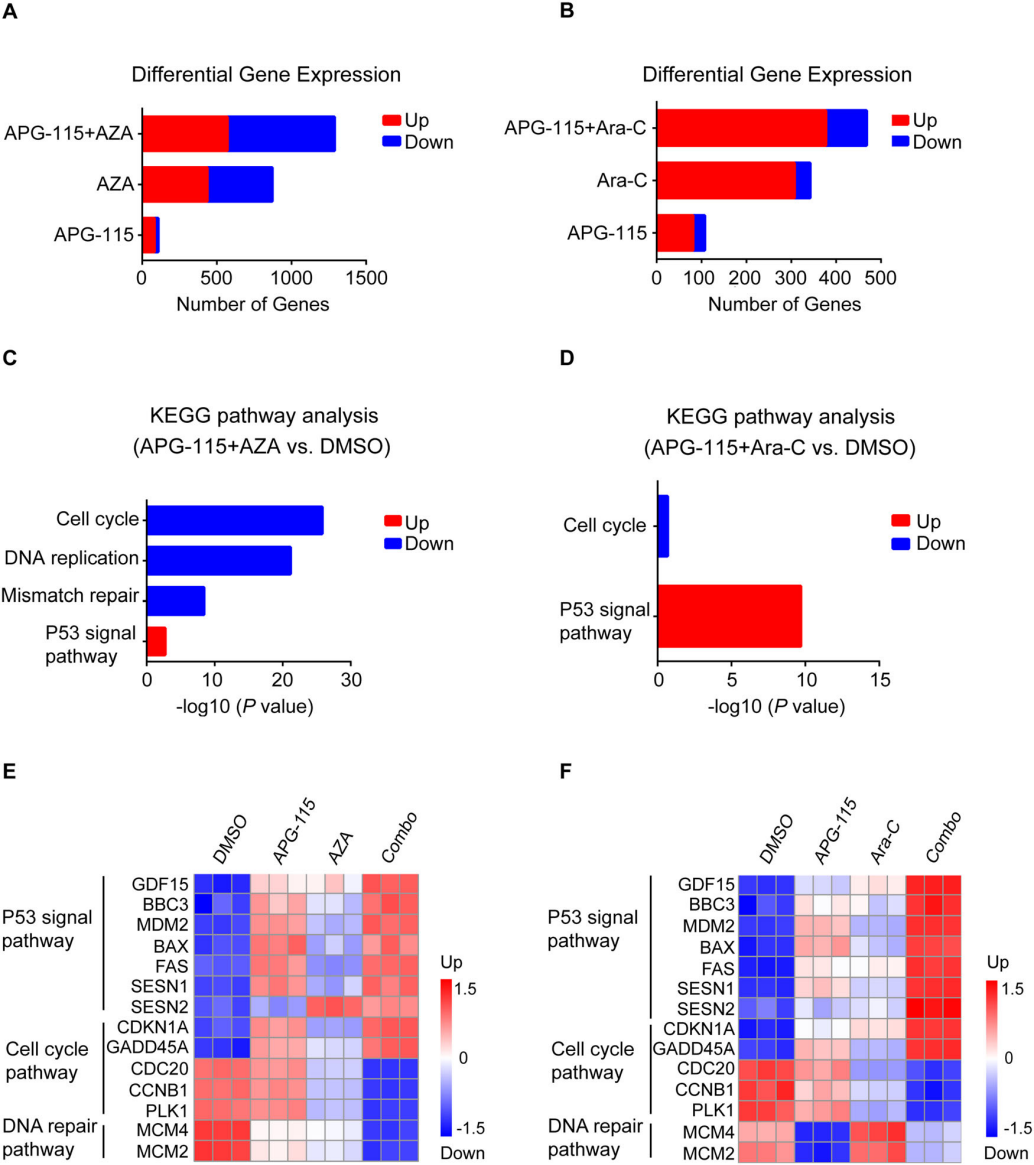
RNA sequence analysis of MOLM-13 cells treated with APG-115, AZA, Ara-C, or combinations as indicated for 24 h. MOLM-13 cells treated with APG-115 (40 nM), AZA (3 μM), Ara-C (100 nM), alone or in combination for 24 h in triplicates. Cells were collected for RNAseq analysis after treatment. **A**, **B** Numbers of differentially expressed genes after each treatment. **C**, **D** The most significantly enriched biological processes upregulated or downregulated in response to combined treatments: both AZA and Ara-C together with APG-115 upregulated P53, while the APG-115-AZA combination more markedly downregulated AML cell cycle, DNA replication, and mismatch repair. **E**, **F** Representative differentially expressed genes in response to APG-115, AZA, or Ara-C, alone or in combination. Combinations of APG-115 with either agent upregulated genes encoding the P53 pathway and downregulated those related to the cell cycle pathway, but only the APG-115-AZA combination markedly downregulated genes implicated in DNA repair. Expression levels are presented as normalized log_2_ counts per million. *BBC3* Bcl-2-binding component 3 gene, *CCNB1* Cdc2-cyclin B1 gene, DMSO dimethylsulfoxide vehicle, *CDC20* cell division cycle 20 gene, *CDKN1A* cyclin-dependent kinase inhibitor 1A gene, *GADD45* growth arrest and DNA damage-inducible 45, *GDF15* growth differentiation factor 15 gene, KEGG Kyoto Encyclopedia of Genes and Genomes, *MCM2/4* minichromosome maintenance proteins 2/4 gene, *PLK-1* serine/threonine-protein kinase (also termed Polo-like kinase 1) gene, *SESN1/2*, sestrin 1 and 2 genes.

Compared to single agents, exposure of MOLM-13 cells to APG-115 combined with AZA or Ara-C resulted in more differentially expressed genes, implicating more marked effects of the combined treatments (Fig. 5A, B). Venn analysis of the intersection and union of differentiated expression genes between single agent and combination treatments are shown in Supplementary Fig. 4. The results showed that the largest number of differentially upregulated and downregulated genes appeared to be induced by the combined treatment with APG-115 and AZA or Ara-C, compared to either single-agent treatments. Interestingly, there are some but not many overlapped targets between the single drug vs. combinations.

According to total RNA sequencing (RNASeq) analysis, exposure of MOLM-13 cells to combinations of APG-115 with AZA or Ara-C caused more marked activation of the P53 pathway and downregulated cell cycle progression and mismatch repair. Our analysis of the Kyoto Encyclopedia of Genes and Genomes (KEGG) demonstrated that the combination of APG-115 with AZA upregulated the expression of genes encoding constituents of the P53 signaling pathway and downregulated those involved in DNA replication, mismatch repair, and cell cycle progression (Fig. 5C). The combination of APG-115 with Ara-C mainly upregulated the expression of genes involved in the P53 pathway while downregulating those involved in cell cycle progression (Fig. 5D).

Upregulated P53-target genes included growth differentiation factor 15 (*GDF15*), which is a surrogate for P53 activation; and apoptogenic gene *BBC3*, which encodes P53 upregulated modulator of apoptosis [PUMA]), *MDM2*, *BAX*, and *FAS*. APG-115 combination treatments also upregulated expression of negative regulators of cell cycle progression genes, including cyclin-dependent kinase inhibitor 1A (*CDKN1A*), which encodes P21; and growth arrest and DNA damage-inducible 45 (*GADD45)*, which plays a role in cell cycle control, DNA repair, and senescence (Fig. 5E, F). Both APG-115 combination treatments upregulated expression of sestrin genes 1 and 2 (*SESN1* and *SESN2*), which are negative regulators of the mammalian target of rapamycin complex-1 (mTORC-1); this complex is, in turn, a sentinel of energy and redox status that regulates protein synthesis. Conversely, APG-115 combinations also significantly downregulated genes that encode oncoproteins involved in cell cycle division and progression, such as *CDC20*, *CCNB1*, and serine/threonine-protein kinase *PLK1*. Notably, treatment with APG-115 in tandem with Ara-C resulted in more substantial increases in P53-regulated genes compared to APG-115 with AZA, whereas only APG-115-AZA markedly reduced DNA replication by downregulating the expression of minichromosome maintenance protein 2/4 (*MCM2*, *MCM4*) genes. Taken together, these findings suggest that each combination of APG-115 with SOC AML treatments exerted antileukemic activity via largely overlapping, but also somewhat distinct, molecular pathways.

### Combinations of APG-115 with DAC, AZA, or Ara-C coordinately induce DNA damage and upregulate expression of P53/P21 in *TP53* wild-type AML cells

**Fig. 6:**
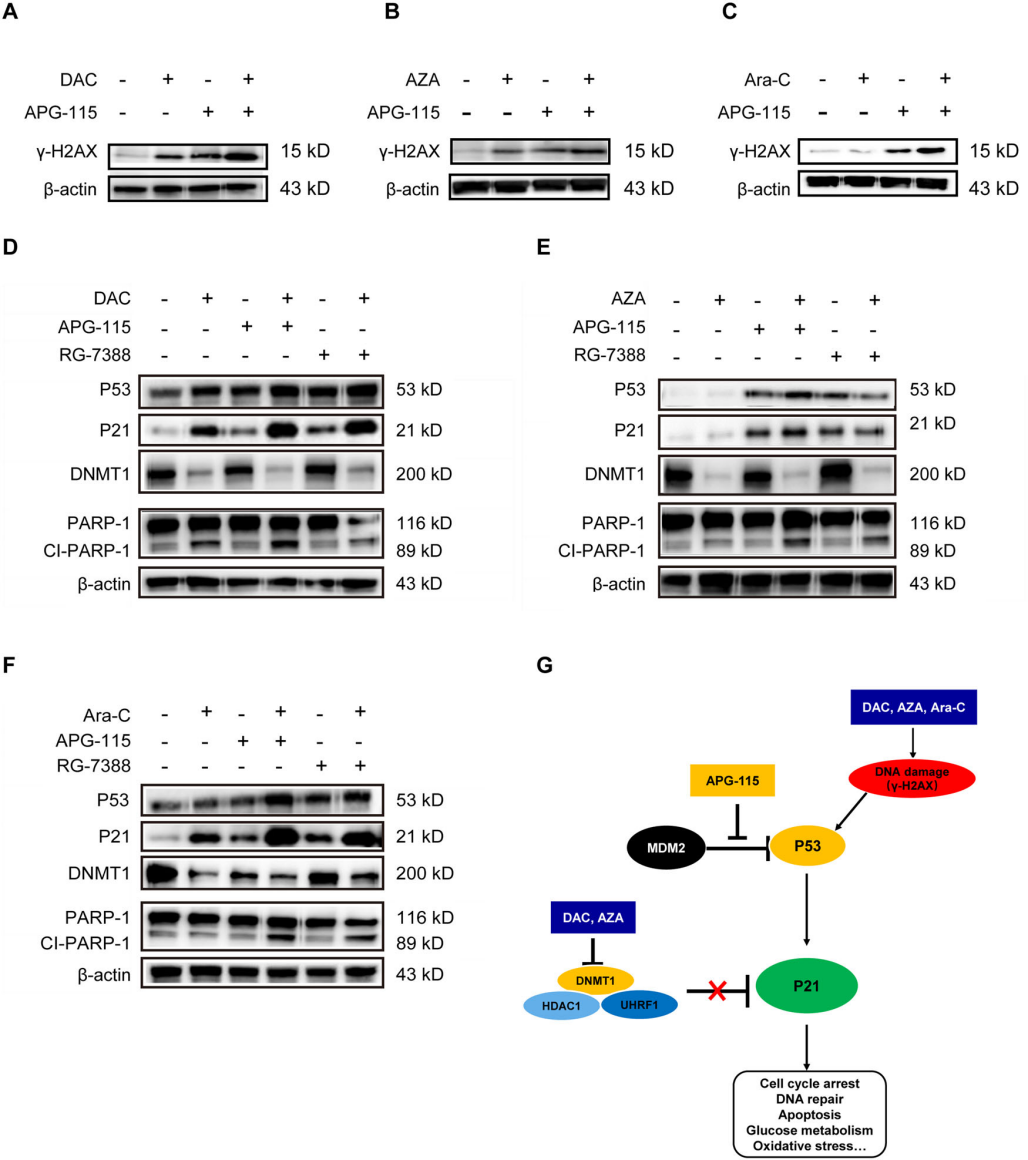
Combined treatment with APG-115 and AZA, DAC, or Ara-C coordinately induces DNA damage and activates the P53/P21 pathway in AML cells. **A**, **B** MOLM-13 cells were treated with DAC (100 nM) or AZA (0.33 µM) for 24 h and then refreshed with DAC or AZA combined with APG-115 (40 nM) for an additional 24 h. Expression levels of γ-H2AX, a biomarker for DNA double-strand breaks, were assessed by Western blot. **C** Expression levels of γ-H2AX in MOLM-13 cells treated with Ara-C (100 nM) alone or in combination with APG-115 (40 nM) for 48 h. **D**–**F** Protein expression in MOLM-13 cells treated with APG-115 (40 nM), AZA (3 µM), Ara-C (100 nM), DAC (100 nM), or RG-7388 (40 nM), or their combinations as indicated for 48 h (**D**, **F**) or 6 h (**E**). β-actin was included as a loading control. Representative results from three independent experiments. **G** Proposed mechanism of action of APG-115 in combination with AZA, DAC, or Ara-C in AML cells. Cl-PARP-1, cleaved poly (ADP‒ribose) polymerase-1; DNMT1, DNA methyltransferase inhibitor; HDAC, histone deacetylase; γ-*H2AX,* H2A histone family member X; UHRF1, ubiquitin-like with PHD and RING finger domains 1.

Further Western blot analyses revealed that combined APG-115 treatments significantly upregulated γ-H2AX in MOLM-13 cells. This histone H2A variant biomarker for DNA damage is indicative of DNA double-strand breaks, which can markedly attenuate genomic stability and compromise cell survival (Fig. 6A–C).

APG-115 treatment also consistently stabilized P53 in MOLM-13 AML cells, leading to accumulation of P53 and P21 proteins (Fig. 6D–F). Perhaps most importantly, P53 and P21 proteins were further upregulated when APG-115 was combined with DAC, AZA, or Ara-C (vs. single SOC agents). Combined with DAC, AZA, or Ara-C, APG-115 also synergistically augmented output of a cleaved 89-kD catalytic fragment of poly (ADP-ribose-1; PARP-1), which is a prominent apoptotic signature. In combination treatments, APG-115 was also more potent than MDM2 inhibitor RG-7388 at the same concentrations. As anticipated, single-agent treatment with AZA or DAC substantially downregulated their key molecular target, DNMT1 protein. On the other hand, Ara-C significantly upregulated the expression of P21 while downregulating the expression of DNMT1.

A putative mechanism underlying the antileukemic activities of APG-115 combination treatments is depicted in Fig. 6G. In brief, APG-115 binds to MDM2 protein and releases its inhibitory effect on P53, activating the P53/P21 pathway by restoring the tumor-suppressor functions of P53. DAC, AZA, or Ara-C cause DNA damage, as evidenced by increased output of γ-H2AX, which further activates P53 and P21 signaling pathways. AZA and DAC also prevent inhibition of the DNMT1–UHRF1–HDAC1 complex on P21^26,27^ and hence contribute to the induction of P21 protein expression. Multidomain ubiquitin-like with PHD and RING finger domains 1 (UHRF1) leads to silencing of gene expression by bridging DNA and histone methylation, while HDAC1 (histone deacetylase 1) modulates P53 effects on apoptosis and cell growth.

## Discussion

In the past four decades, bone marrow transplantation and chemotherapy have predominated the AML treatment landscape. Chemotherapy, especially the 7 + 3 regimen, continues to be the induction therapy of the first choice for many patients with newly diagnosed AML. Since 2017, several targeted therapeutics have been introduced to the AML treatment armamentarium. Despite these notable advances, the average life expectancy of patients with AML has not appreciably increased in recent years^28,29^. Because of the overall genetic and clinical heterogeneity of AML, meeting the critical unmet need of enhancing patient outcomes will require additional, novel, effective, and well-tolerated treatments.

A highly attractive treatment strategy for AML is to inhibit MDM2, restore the native tumor-suppressing functions of P53, and induce P53-dependent apoptosis in malignant cells. Preclinical and clinical studies of several MDM2 inhibitors have supported the viability of this approach to mediate antileukemic activity^30–33^.

Against this backdrop, we evaluated the ability of novel MDM2 inhibitor APG-115 to disrupt MDM2-P53 interactions in *TP53*^*wt*^ AML. Not only did APG-115 exhibit antiproliferative cellular activity and induce both apoptosis and G_0_/G_1_ cell cycle arrest; it potentiated antileukemic effects when combined with SOC treatments in AML xenograft models. In the systemic MOLM-13 model, APG-115 augmented median survival by 18.5 days compared to vehicle control and significantly (by 2–6 days) compared to MDM2 inhibitor RG-7388.

APG-115 induced accumulation of P53 and its transcriptional target P21 protein in our study. Combining AZA, DAC, or Ara-C with APG-115 synergistically potentiated its P53/P21-activating effects, which can most likely be ascribed to shared effects of these agents on the same targeted signaling pathways. In this context, activation of the P53/P21 pathway restores tumor suppression function, leading to more profound DNA damage and apoptosis. However, the synergistic effects of APG-115 combination treatments could not be attributed to increased cell cycle arrest.

Intriguingly, MOLM-13 and MV-4-11 AML cell lines carrying *TP53*^*wt*^ and concurrent *FLT3-ITD*^*mut*^ mutations were the most sensitive to APG-115-induced growth inhibition, compared to non-*FLT3*-mutated OCI-AML-3 cells. In addition, the MOLM-13 CDX model is a diffuse systemic tumor with a high degree of malignancy and disease progression. Therefore, our data demonstrate that APG-115 as a single agent is highly effective in reducing the leukemic burden and prolonging survival of mice bearing AML in vivo, and *FLT3*^*mut*^ AML cells may be more sensitive to MDM2 inhibitors compared to wild-type *FLT3*.

Evidence from the present study also suggests that the antileukemic activity of APG-115 is more potent than RG-7388 both in vitro and in vivo. When combined with SOC agents, APG-115 induced apoptosis more markedly, as evidenced by increased cleavage of PARP-1 protein. Unknown at this juncture is whether these promising but preliminary findings with APG-115 can be extended to patients with AML. To address this question, multiple clinical trials of APG-115 and other MDM2 inhibitors combined with SOC agents have been undertaken in patients with AML and MDS^32^. A phase Ib, open-label, 3 + 3 dose-escalation study will evaluate the safety (dose-limiting toxicities), pharmacokinetics, and pharmacodynamics of APG-115, alone or combined with AZA or Ara-C, in patients with r/r AML and relapsed and high-risk MDS (NCT04275518).

Other clinical trials of MDM2 inhibitors in patients with AML are ongoing (clinicaltrials.gov). Despite being the focus of ongoing clinical inquiry to assess potentially synergistic antitumor effects, these agents have unclear complementary mechanisms. Both AZA and DAC are DNA hypomethylating agents with previously reported apoptosis-inducing effects in AML cells^34^. Each agent acts chiefly by depleting DNMT1 and inducing DNA hypomethylation and damage^34^. DNMT1 forms complexes with UHRF1 and HDAC1 proteins in tumor cells, reducing expression of tumor suppressor promoter genes (e.g., *P16INK4A*, *P14ARF*, and *P21*) and hence fostering tumor growth^26,27^. In this context, activation of the P53-mediated apoptosis pathway has been implicated in DAC-induced cell death^35^. Ara-C is a pyrimidine nucleoside analog that induces DNA damage, activating the ATM/ATR-P53/P21 signaling cascade, inducing cell cycle arrest, and markedly impeding cellular proliferation^36^. These combinations of DNA hypomethylating, and cytotoxic, agents may act via the P53/P21 signal-transduction pathway.

One other potentially viable combination of APG-115 is with immuno-oncologic (I-O) therapies. In addition to promoting the intrinsic (mitochondrial) pathway of apoptosis, APG-115 exhibits potential immunomodulatory properties, according to our previous preclinical research^37^. Future studies might assess the availability of appropriate AML models in immunocompetent animals. Potential immunomodulatory effects of APG-115 in tandem with the PD-1 inhibitor pembrolizumab are being evaluated in an ongoing phase I trial of patients with unresectable or metastatic melanoma or advanced solid tumors (NCT03611868).

Hematological toxicity due to on-target myelosuppression by continuous MDM2 inhibition remains a challenge for MDM2-targeted treatment in clinic^38^. However, myelosuppression is manageable in the clinic. An important approach to mitigate the toxicity is to optimize the doses and dosing schedules. Various doses and schedules were evaluated in order to achieve the optimal balance between efficacy and toxicity in the preclinical setting^39,40^. It is interesting to unveil that the high-dose pulse of an MDM2 inhibitor led to a rapid onset of apoptosis^40^. Consistently, a high-dose pulse daily dosing of RG7388 for 5 days demonstrated antitumor activity equivalent to chronic administration^39^. Accordingly, the application of a high-dose pulse schedule of RG7388 achieved clinical responses with manageable toxicity in patients with AML^25^. Such a 5- to 7-day high-dose pulse schedule was successfully adapted in multiple clinical trials to evaluate MDM2 inhibitors in cancer patients (clinicaltrails.gov).

In conclusion, our study affords consequential new insights into the in vitro and in vivo tumor-suppressing effects of the novel MDM2 inhibitor APG-115, alone and in concert with SOC agents, in AML cells and animal models. We have formulated plausible molecular mechanisms underlying the synergistic effects of our MDM2 inhibitor in tandem with DNA-hypomethylating and DNA-damaging agents in AML, including P53 and downstream signaling pathways. These data support further, clinical development of APG-115, especially in combination with these SOC agents, to enhance AML management. In this connection, phase I/II clinical trials of APG-115 combined with SOC agents in AML are underway (NCT04358393).

## Materials and methods

### Cell lines and reagents

Human AML cell lines, including MV-4-11, MOLM-13, and HL-60, were purchased from American Type Culture Collection (ATCC; Manassas, VA, USA). Human AML cell line Ontario Cancer Institute‒Acute Myeloid Leukemia-3 (OCI-AML-3) was sourced from Cobioer (Nanjing, China) and SKM-1 from the Japanese Collection of Research Bioresources Cell Bank (JCRB). MV-4–11 cells were propagated in Iscove’s Modified Dulbecco’s Medium (IMDM, Gibco, Grand Island, NY, USA Cat# C12440500BT) supplemented with 10% fetal calf serum (FCS, AUSGENEX, Loganholme, QLD, Australia, Cat# FBSSA500-S). Other cell lines were propagated in Roswell Park Memorial Institute (RPMI) 1640 medium (Gibco, Cat# C11875500BT) containing 10% FCS. All experiments utilized genetically authenticated, microbial-free cells in their exponential phases of growth.

APG-115 (produced by Ascentage Pharma, Jiangsu, China) was dissolved in dimethyl sulfoxide (DMSO, Sigma, St. Louis, MO, USA, Cat# D8418) for in vitro experiments or suspended in 0.2% hydroxypropyl methylcellulose (HPMC, Sigma, Cat# H7509-25G) for in vivo animal studies. DAC (Cat# S120009), AZA (Cat# S178206), and Ara-C (Cat# S164806) purchased from Selleckchem were dissolved in DMSO for in vitro assays and saline for in vivo studies. RG-7388 (idasanutlin, MDM2-P53 inhibitor, MedChemExpress, Cat# HY-15676) was dissolved in DMSO for in vitro assays.

### Cell viability assay

AML cells were seeded at a density of 8000 cells in an opaque 96-well plate and incubated with increasing concentrations of APG-115, AZA, DAC, or Ara-C, or with vehicle (DMSO). After treatment, cell viability was measured by Promega^®^ Cell-Titer Glo^®^ 3D Cell Viability Assay luminescence assay kit according to instructions from the manufacturer (Promega, Madison, WI, USA, Cat# G7571). Luminescence signal was detected using a BioTek Synergy H1 Hybrid Multi-Mode (Microplate) Reader (BioTek, Shanghai).

To assess the synergy of combination regimens for AML, we used CalcuSyn software (v2.0, Biosoft, Cambridge, UK), which allowed us to compute values for half-maximal inhibitory concentration (IC_50_) and the combination index (CI). A CI value of less than 0.9 indicates a synergistic effect; a CI value equal to 0.9 indicates an additive effect, and a CI value exceeding 0.9 indicates an antagonistic effect^41^. Cellular proliferative (i.e., viability) curves were plotted using Graphpad Prism version 6.0 software (GraphPad Software, San Diego, CA, USA).

### Cell cycle and apoptosis analysis

For cell cycle analysis, we exposed *TP53* wild-type AML cell lines to APG-115, DAC, AZA, or Ara-C, either alone or combined, for 48 h. After treatment, cells were harvested and fixed with ice-cold 70% (v/v) ethanol (General reagent, Cat# G7353N) for 24 h and centrifuged at 200×*g* for five minutes. Cell pellets were then washed once with phosphate-buffered saline (PBS) and stained with propidium iodide (PI) using a cell cycle analysis kit (Fcmacs, Cat# FMS-CCC01) according to manufacturer instructions. A total of 20,000 events were acquired and proportions of cells in each phase of the cell cycle were calculated using FlowJo^™^ software v10.4.2 (BD Biosciences; San Jose, CA, USA).

For apoptosis analysis, AML cells were treated with APG-115, DAC, AZA, Ara-C, alone or in combination, for 48 h, then subjected to flow cytometry analysis using an Annexin V/fluorescein isothiocyanate (FITC)/PI (Annexin V-FITC-PI) Apoptosis Detection Kit (BD Biosciences Cat# 556547). In brief, cells were washed once with cold PBS and incubated for 15 min at room temperature with Annexin V-FITC-PI in binding buffer. Cells were then analyzed on an Attune NxT Flow Cytometer (Life Technologies, Thermo Fisher, Carlsbad, CA, USA) using FlowJo software. Results were expressed as percentages of Annexin V^+^ cells. Experiments were performed in duplicate three independent times.

### Human CD45^+^ cell staining and analysis by flow cytometry

Bone marrow cells from two femurs and spleen cells were collected and lysed in 2 mL 1× red-blood-cell lysis buffer (BD Biosciences, Cat# 555899) at room temperature for 2 min, followed by centrifugation at 1000 rpm for 5 min. Supernatants were decanted and discarded. Cell pellets were washed twice with PBS, followed by dark incubation with live/dead antibody (eBioscience, San Diego, CA, USA, Cat# L34976) for 15 min. Cells were then washed twice with PBS and incubated with human CD45-APC antibody (BD Biosciences, Cat# 555485) diluted in Magnetic-Activated Cell Sorting (MACS^®^) buffer (Miltenyi Biotec, Auburn, CA, USA, Cat# 130-091-221) at 1:5 for 30 min on ice. The buffer consisted of PBS (pH 7.2), 0.5% bovine serum albumin, and 2 mM EDTA diluted in MACS^®^ bovine serum albumin stock solution with autoMACS^®^ rinsing solution. After incubation, cells were washed twice with PBS and processed for flow cytometry analysis on the Attune NxT Flow Cytometer. Data were analyzed using FlowJo software and reported as percentages of human CD45^+^ cells within live cells.

### Western blot analysis

As described previously^37^, cells were collected and lysed in radioimmunoprecipitation assay lysis buffer (Beyotime, China, Cat# P0013B) containing a 1% protease inhibitor cocktail (Yeasen, China, Cat# 20124ES10), 1% InStab^TM^ phosphatase inhibitor cocktail (Yeasen, Cat# 20109ES05), and 1% phenyl-methyl-sulfonyl fluoride (PMSF Yeasen, Cat# 20104ES08). Protein concentration was quantitated using a bicinchoninic acid protein assay kit (Beyotime, Cat# P0010).

Equal amounts of soluble protein were loaded and separated using 4–20% sodium dodecyl sulfate-polyacrylamide gel electrophoresis (GenScript, Cat# M42010C), followed by transfer to a polyvinylidene difluoride membrane (Millipore, MA, USA, Cat# IPVH00010) and then exposed to immunoblotting antibodies against P21 (CST, MA, USA, Cat# 2947S), P53 (CST, Cat# 2524S), poly (ADP-ribose) polymerase (PARP-1, Cat# 9532S), DNA methyltransferase (DNMT1, CST, Cat# 5032S), mouse double-minute 2 homolog (MDM2, CST, Cat# 86934S), anti-γ-H2AX (CST, Cat# 2577S), and β-actin (CST, Cat# 4970S). Antirabbit or antimouse horseradish peroxidase-conjugated secondary antibodies purchased from Yeasen (Cat# 33101ES60 and 33201ES60) were used at 1:5000 dilution. Immunoreactive proteins were visualized using an Azure c300 Chemiluminescent Western Blot Imaging System (Azure Biosystems, Dublin, CA, USA). Western blots were at least repeated twice to ensure reproducibility.

### RNA sequencing

MOLM-13 cells were treated for 24 h. Total RNA was extracted from 1 × 10^6^ cells using Invitrogen TRIzol^®^ RNA Isolation Reagent (Thermo Fisher, MA, USA, Cat# 15596018) and RNeasy MinElute spin columns based on silica-membrane technology according to manufacturer’s instructions (Qiagen, Cat# 74204, Germantown, MD, USA). The integrity of the total RNA was assessed using a 2100 Bioanalyzer Instrument (Agilent, Santa Clara, CA USA) and quantitated using NanoDrop^®^ (Thermo Scientific). A total of 500 ng of a high-quality RNA sample was used to construct a sequencing library. RNA quality was determined according to an optical density [OD] 260/280 nm of 1.9–2.0 and an RNA integrity number [RIN] of at least 8, where integrity is graded from 1 (most degraded) to 10 (least degraded).

Messenger RNA-focused sequencing libraries from total RNA were prepared using a TruSeq mRNA Library Prep kit (Illumina, Cat RS-122-2001, San Diego, CA USA). Sequencing was performed using this system according to protocols provided by the manufacturer for 2 × 150 base-pair paired-end sequencing at WuXi NextCODE (Shanghai). Reads were aligned to a reference genome by using Spliced Transcripts Alignment to a Reference (STAR) software^42^ with default parameters. Differential expression analysis was performed using the edgeR package in R (Bioconductor, Stanford, CA, USA)^43^. Criteria for significantly differentially expressed genes comprised a false-discovery rate of less than 0.05 and a |fold change| of at least two. A set of significantly upregulated or downregulated genes was inputted to test the significance of gene enrichment in a KEGG (https://www.genome.jp/kegg/) path by using GOstat packages in R. The criteria value was *p* < 0.01.

### AML xenograft models

All experiments were conducted in the animal facility of GenePharma (Suzhou, China). Protocols and experimental procedures involving the care and use of animals were approved by the GenePharma Institutional Animal Care and Use Committee. Animals could acclimate to the environment for at least three days then transferred to a temperature- (20–26 °C) and humidity-controlled (40–60%, relative humidity) SPF room with a 12-h light/12-h dark cycle during the experimental period. Animals were housed in cages, with no more than five mice per cage. Animals had free access to sterile drinking water and food.

For the systemic AML model, 6- to 8-week-old female nonobese diabetic severe combined immunodeficient mice (NOD SCID) were pretreated with cyclophosphamide (150 mg/kg, intraperitoneally) for two consecutive days. The MOLM-13 systemic AML xenograft model was then established by intravenous inoculation of 1 × 10^7^ cells. After 3 days, tumor-bearing mice were randomized to treatment with vehicle, APG-115, or AZA, alone and in combination. Mice were monitored daily for the development of hind-limb paralysis or abdominal swelling resulting from disease progression, as well as more than a 20% body weight loss. These clinical manifestations served as humane endpoints.

For the subcutaneous model, OCI-AML-3 cells (1 × 10^6^) were injected subcutaneously into the right flanks of six- to eight-week-old nonobese diabetic severe combined immunodeficient (NOD SCID) mice. Approximately, 10 days after cellular inoculation, mice were randomly grouped based on primary body weight and tumor volume, which reached a mean of approximately 100–150 mm^3^. APG-115, AZA, and DAC were then administered alone or in combination. Tumor volumes were measured by caliper twice weekly and expressed in mm^3^ using the following formula:

1 $${\mathrm{Tumor}}\;{\mathrm{volume}}\;\left( {{\mathrm{mm}}^3} \right) = \left( {{a}\; \times \;{b}^2} \right)/2$$

where *a* was the long diameter of the tumor and *b* was the short diameter. Mice were humanely euthanized when their body weight loss >20% or a subcutaneous tumor volume reached 2000 mm^3^.

As a measurement of efficacy, a treatment/control (T/C%) value was calculated as the mean RTV of treated tumors (T) divided by the mean RTV of control tumors (C) × 100%, where T_RTV_ was the relative tumor volume (RTV) of the treatment group and C_RTV_ was the RTV of the control group. RTV is expressed as the ratio *V*_t_/*V*_1_, where *V*_t_ is the average tumor volume at a certain time point (Day *t*) and *V*_1_ is the average tumor volume on the first day of treatment (Day 1).

2 $${\mathrm{Tumor}}\;{\mathrm{growth}}\;{\mathrm{inhibition}}\;\left( {{\mathrm{TGI}}} \right)\;{\mathrm{was}}\;{\mathrm{calculated}}\;{\mathrm{as}}\;\left( {1 - {\mathrm{T}}/{\mathrm{C}}\% } \right) \times 100\% .$$

### Statistical analysis

One-way analysis of variance, followed by Games–Howell’s posttest to adjust for multiple comparisons^44^, was conducted to assess the statistical significance of differences between multiple treatment groups. In the MOLM-13 systemic AML model, the date of the last mouse death was used to analyze median overall survival, with the generation of Kaplan–Meier plots. Survival curves of different treatment groups were compared using the log-rank test with Bonferroni’s test for multiple comparisons. All data were analyzed in SPSS version 18.0 (IBM PASW Statistics for Windows; Chicago, IL USA). GraphPad Prism version 6.0 was used for graphic presentation.

## Supplementary information

Supplementary Table 1

Supplementary Figure 1

Supplementary Figure 2

Supplementary Figure 3

Supplementary Figure 4

Supplementary figure and table legends

## Data Availability

The datasets used and/or analyzed during the current study are available from the corresponding author on reasonable request.
