# Supplementary material for: MDM2 inhibitor APG-115 exerts potent antitumor activity and synergizes with standard-of-care agents in preclinical acute myeloid leukemia models
Source: Cell Death Discov. 2021 May 3;7:90. doi: 10.1038/s41420-021-00465-5 (PMC8093284; doi:10.1038/s41420-021-00465-5)
Supplement: Supplementary file 1 — Supplementary Table 1 [file 41420_2021_465_MOESM1_ESM.docx]

| **Cell line** | ***TP53*** | ***FLT3*** | ***NPM1*** | ***RAS*** | **IC_50_, nM (Mean ± SD, n=2)** | |
| --- | --- | --- | --- | --- | --- | --- |
|  |  |  |  |  | **APG-115** | **RG-7388** |
| MOLM-13 | wt | ITD | wt | wt | 26.8 ± 4.9 | 73.2 ± 14.1 |
| MV-4-11 | wt | ITD | wt | wt | 165.9 ± 42.4 | 779.1 ± 351.6 |
| OCI-AML-3 | wt | wt | mut | mut | 315.6 ± 97 | NA |
| HL-60 | del | wt | wt | wt | 2,558.3 ± 581.5 | NA |
| SKM-1 | mut | wt | wt | wt | 8,947.3 ± 569.6 | >10,000 |
