# Supplementary figures and images for: MDM2 inhibitor APG-115 exerts potent antitumor activity and synergizes with standard-of-care agents in preclinical acute myeloid leukemia models
Source: Cell Death Discov. 2021 May 3;7:90. doi: 10.1038/s41420-021-00465-5 (PMC8093284; doi:10.1038/s41420-021-00465-5)

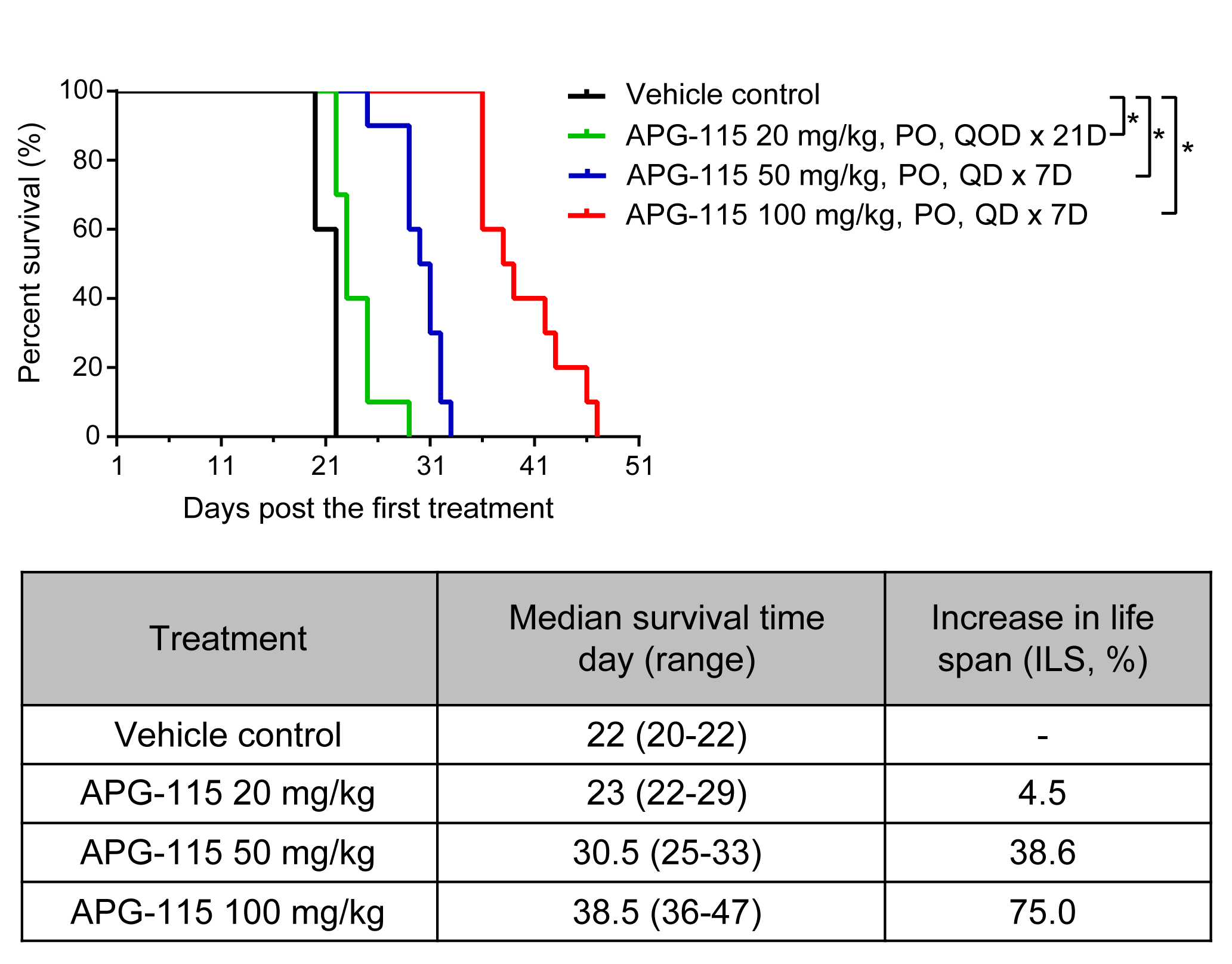

Supplement: Supplementary file 2 — Supplementary Figure 1 [file 41420_2021_465_MOESM2_ESM.tif]

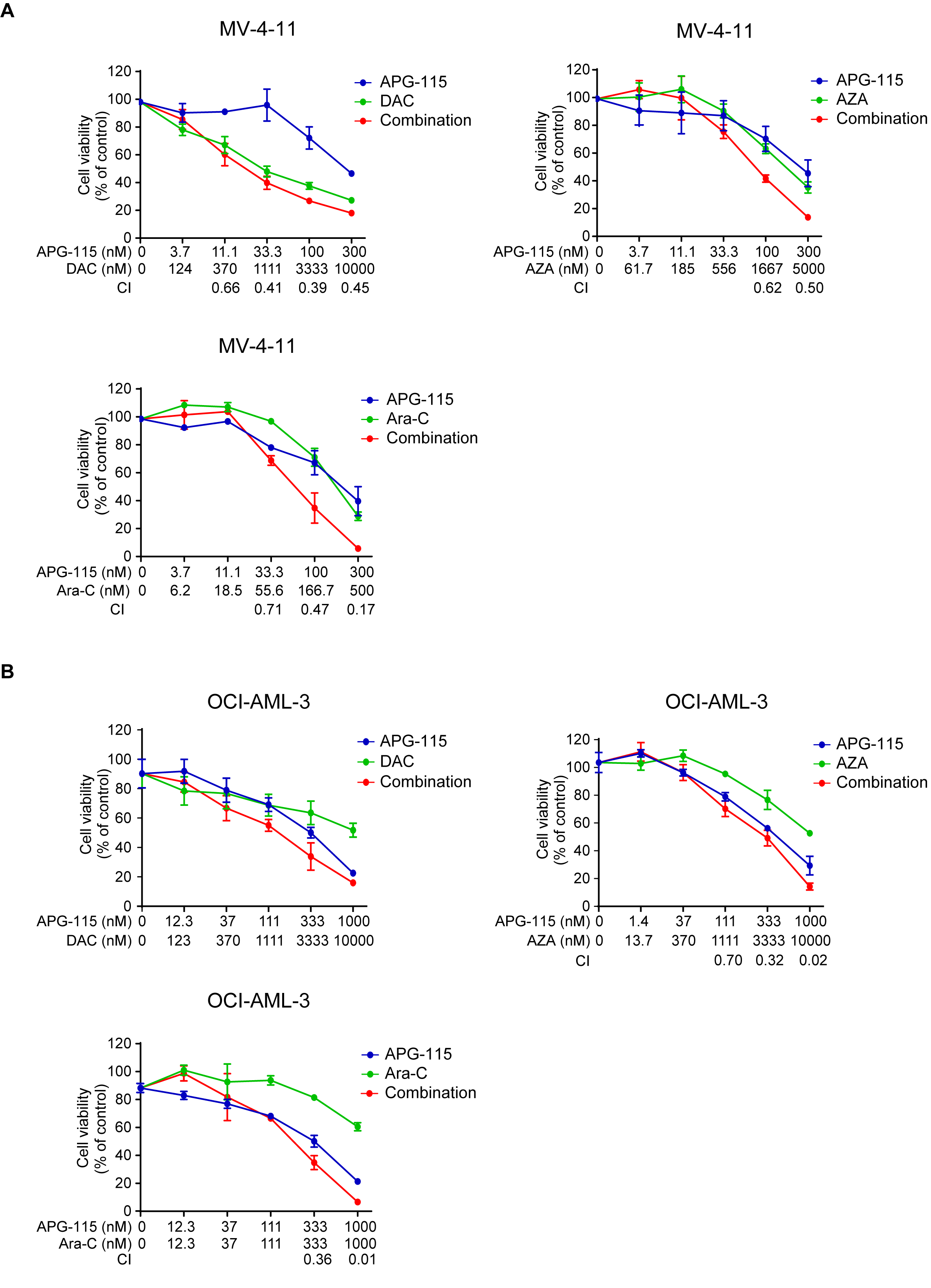

Supplement: Supplementary file 3 — Supplementary Figure 2 [file 41420_2021_465_MOESM3_ESM.tif]

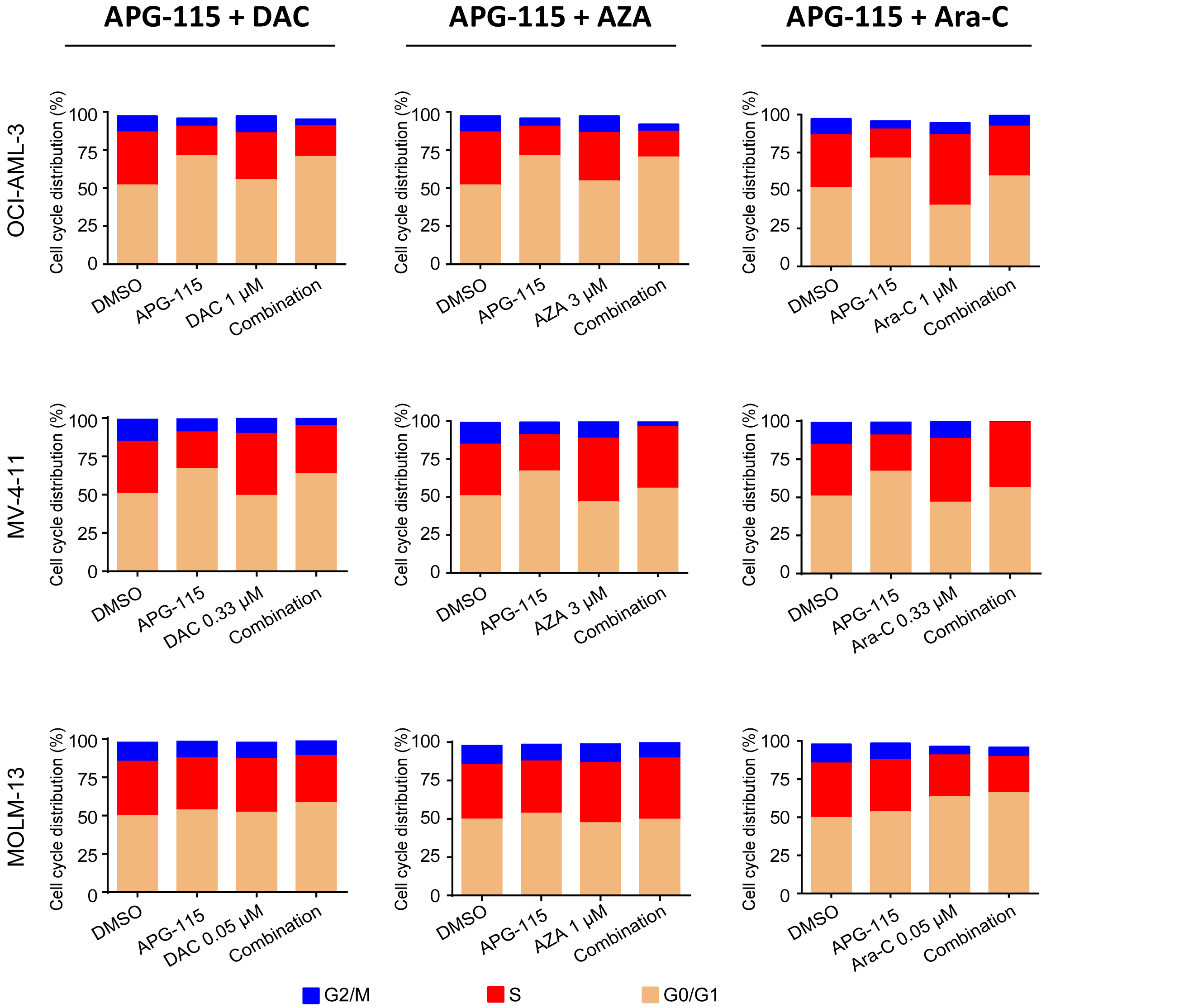

Supplement: Supplementary file 4 — Supplementary Figure 3 [file 41420_2021_465_MOESM4_ESM.tif]

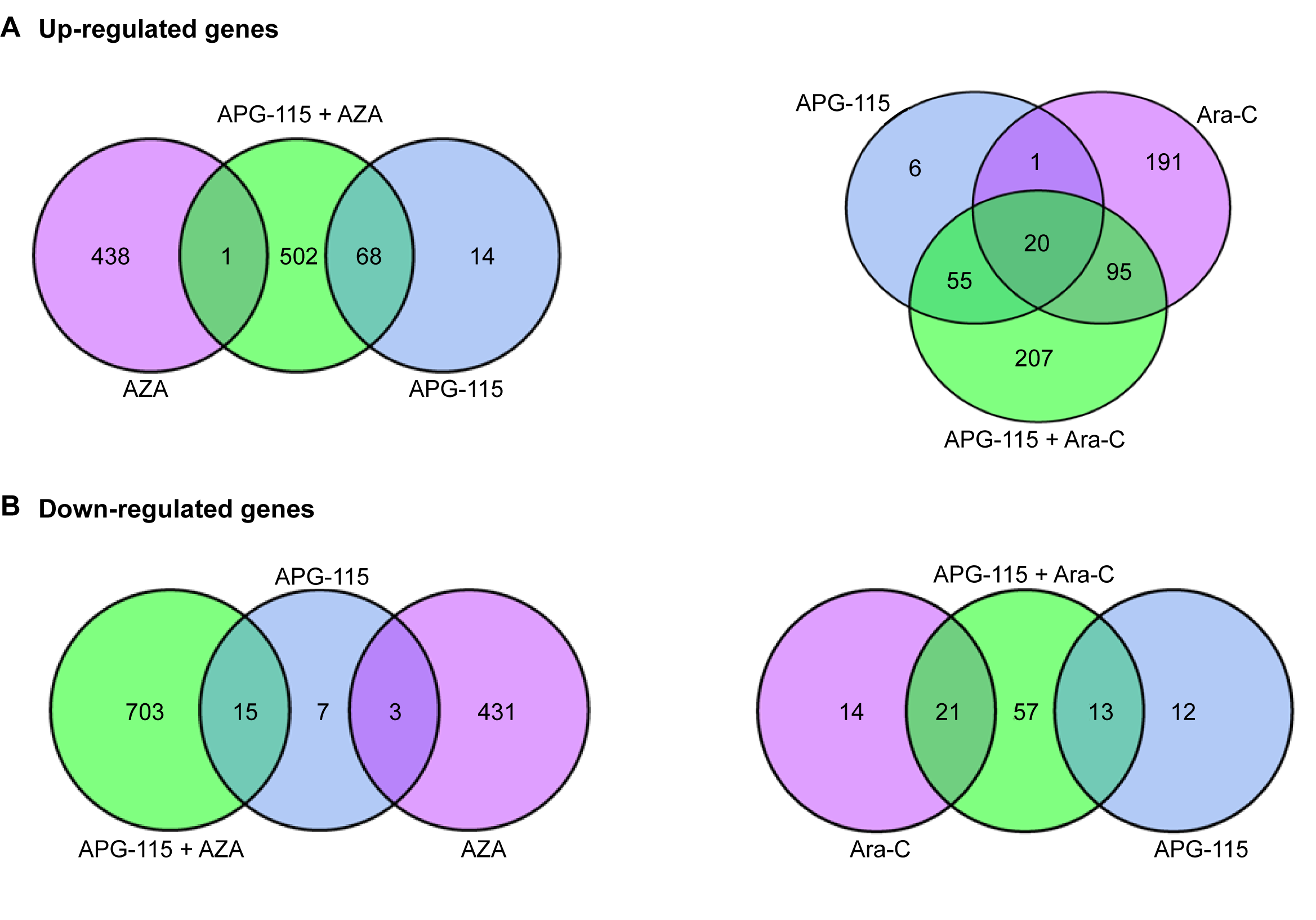

Supplement: Supplementary file 5 — Supplementary Figure 4 [file 41420_2021_465_MOESM5_ESM.tif]
