# Supplementary material for: MDM2 inhibitor APG-115 exerts potent antitumor activity and synergizes with standard-of-care agents in preclinical acute myeloid leukemia models
Source: Cell Death Discov. 2021 May 3;7:90. doi: 10.1038/s41420-021-00465-5 (PMC8093284; doi:10.1038/s41420-021-00465-5)
Supplement: Supplementary file 6 — Supplementary figure and table legends [file 41420_2021_465_MOESM6_ESM.docx]

**Supplementary Table. 1 Antiproliferative activity of APG-115 in AML cell lines exhibiting different genetic variants**

del, deletion; wt, wild type; *FLT3*, fms-like tyrosine kinase 3 gene; HL-60, human leukemia cell line; IC_50_, half-maximal inhibitory concentration; ITD, internal tandem duplications; mut, mutant; NA, not available; *NPM1*, nucleophosmin (nucleolar phosphoprotein B23; numatrin) gene; OCI-AML-3, Ontario Cancer Institute‒Acute Myeloid Leukemia-3 cell line. MDM2 inhibitor RG-7388 was included as a reference.

**Supplementary Fig. 1 Treatment with single-agent APG-115 significantly prolongs survival in a systemic AML xenograft model derived from *TP53^wt^* MOLM-13 cells.** Nonobese diabetic severe immunodeficient mice intravenously implanted with 1×10^7^ MOLM-13 cells (n=10/group) received vehicle, APG-115 at 20 mg/kg (PO, QOD for 21 days), 50 mg/kg (PO, QD for 7 days), or 100 mg/kg (PO, QD for 7 days) 3 days after cell implantation. Kaplan-Meier curve depicting mouse survival. ^*^*p* < 0.05.

**Supplementary Fig. 2 aPG-115 synergizes with AZA, DAC, and Ara-C to inhibit cell proliferation in *TP53^wt^* MV-4-11 and OCI-AML-3 AML cell lines.** MV-4-11 (**A**) and OCI-AML-3 cells (**B**) were treated with increasing concentrations of APG-115, AZA, DAC, or Ara-C, alone or in combinations, for 72 hours. Cell viability was determined using the Cell-Titer Glo® 3D Cell Viability Assay luminescence assay kit according to instructions from the manufacturer (Promega). Combination index was computed using CalcuSyn software (v2.0, Biosoft, Cambridge, UK). A CI value of less than 0.9 indicates a synergistic effect.

**Supplementary Fig. 3 aPG-115 in combination with AZA, DAC, or Ara-C dose not further enhance cell cycle arrest in AML cell lines.** AML cells were treated with APG-115 (0.33 µM) and indicated concentrations of AZA, DAC, and Ara-C, alone and in combinations, as indicated for 48 hours. Percentages of cell cycle phases were determined by flow cytometry.

**Supplementary Fig. 4 Differential gene expression by Venn diagram analysis.** MOLM-13 cells treated with APG-115 (40 nM), AZA (3 μM), Ara-C (100 nM), alone or in combination for 24 hours in triplicates. Cells were collected for RNAseq analysis after treatment. **A** Venn diagram showing the overlap of up-regulated genes after treatments. **B** Venn diagram showing the overlap of down-regulated genes after treatments.
